# Evolution of Teachers’ Teaching Beliefs About Mathematics in a Teacher Education Program

**DOI:** 10.3390/bs14100934

**Published:** 2024-10-11

**Authors:** Dandan Sun, Qiaoping Zhang

**Affiliations:** 1 School of Mathematics and Statistics, Shandong Normal University, Jinan 250358, China; ddsun@sdnu.edu.cn; 2 Department of Mathematics and Information Technology, The Education University of Hong Kong, Hong Kong 999077, China

**Keywords:** mathematics teacher, teaching beliefs, development of teacher beliefs, case study

## Abstract

Teachers’ teaching beliefs encompass the underlying perspectives that they hold. These beliefs significantly influence teacher actions in complex ways, thereby impacting the implementation of curriculum reform. However, existing research has revealed inconsistencies between teaching beliefs and actual practices. Employing a case study approach, this study adopts a reflective belief model to address this issue and explore the mechanisms of teaching belief formation within the context of a teacher education program in China. The findings indicate that teaching beliefs can evolve and develop through five dimensions: justification, exemplification, consistency, connection, and practicality. By considering these dimensions within the framework of the reflective belief model, we can better comprehend the inconsistencies between belief and practice. Teacher educators can utilize this understanding to foster the cultivation of reflective teaching beliefs for teachers within training programs.

## 1. Introduction

Beliefs, defined as psychologically held premises or propositions deemed to be true by individuals [1], can significantly influence teachers’ thoughts, actions, and teaching practices [2]. Among these, teaching beliefs—encompassing student views, teaching goals, roles, instructional approaches, and outcomes—most directly impact teaching practice [2]. However, research has exposed an inconsistency between teachers’ stated teaching beliefs and their actual practices, with reform-oriented beliefs often yielding to more traditional methods [3,4,5].

Two categories of factors may inhibit teachers from effectively implementing their teaching beliefs [6,7]. The first category comprises external factors. For instance, a school’s culture that focus on standardized testing, curricular mandates, or a lack of support for innovative teaching methods or pressure from administrators and parental expectations can sometimes conflict with a teacher’s individual teaching beliefs, complicating their incorporation into classroom practice [8,9,10]. The second category involves internal factors. Teachers without the specific skills or knowledge cannot implement their teaching beliefs effectively [11]. Teachers’ beliefs about their own capabilities can also influence how they implement their teaching beliefs [12]. If teachers doubt their abilities to execute certain teaching methods successfully, they may refrain from trying them. However, some researchers have posited that the inconsistency is perceptual, existing from a researcher’s viewpoint but not from the teacher’s perspective [1,13]. This position calls for more exploration of how beliefs are held, which could offer a deeper understanding of teachers’ beliefs and the divergence between teaching beliefs and practices [14,15]. In mainland China, curriculum reform has been progressing. Since the 21st century, the new curriculum concept is mainly reflected in two versions of the Mathematics Curriculum Standard. It is advocated that the teaching should aim to stimulating students’ interest in learning and foster their mathematical thinking. Teachers are encouraged to move away from a lecture-based approach and instead focus more on inquiry, participation, and interaction so that students have enough time and space to engage in activities such as observation, conjecture, and reasoning [16,17]. From the perspective of educational philosophy, learner-focused teaching is preferred over content-focused teaching [18,19]. The new curriculum also states that the course content should include not only the results of mathematics, but also the process of the formation of mathematical results. The students should understand the origin and source, structure and correlation, and value and significance of mathematical knowledge [16,17]. Here, emphasis is on conceptual understanding instead of mastery of mathematical rules and procedures [18,19]. In light of the new curriculum requirements, what beliefs do teachers hold, and what teaching practices do they adopt? This study explores the relationship between teachers’ teaching beliefs and practice, focusing on how these beliefs were held. It also examines the evolution of teaching beliefs, revealing how certain teaching practices promoted by the new curriculum align with different ways in which these beliefs are maintained. Using a case study methodology, this paper addresses three primary research questions:

(i) What were the case teachers’ pre-existing beliefs about mathematics teaching, and how were these beliefs held?
(ii) From the perspective of the way beliefs were held, how can we explain the relationship between teachers’ beliefs and practices before the program?
(iii) How did the ways these beliefs were held develop due to teacher education programs?

## 2. Conceptual Framework

There is a consensus that one’s beliefs about teaching impact how one teaches [20], adopting Rokeach’s description, as follows: All beliefs are predispositions to action [21] (p. 113). However, not all beliefs are held similarly by the belief holder. How beliefs are held may influence their impact on practice. Beliefs ascribed greater importance or possessing greater psychological centrality may significantly influence practice [1,6]. Conversely, peripheral beliefs with lower priority might be difficult to implement in practice. Green systematically highlighted the often-overlooked distinction between what we believe and how we believe it [22].

The psychological strength of a belief is determined by its relationship with other beliefs, or its coherence with other beliefs [13]. Beliefs never appear in complete isolation. When someone adopts a new belief, it connects with other beliefs, collectively supporting each other and forming a part of the larger structure of their belief system [23,24]. Beliefs often relate to each other in a reason–conclusion logic, with the holder’s subjectivity playing a role in the quasi-logical structure [22]. This reason–conclusion logical relationship can be used to justify the rationality of related beliefs. The more relationships there are, the more central the beliefs may be. In addition to quasi-logically related beliefs, beliefs can also be attributed to knowledge, personal experiences, and other forms of evidence [22]. Unlike beliefs that we ‘just believe’, evidence like knowledge and experience are things that we ‘more than believe—we know’ [13]. If a belief system is likened to a raft floating on the water rather than a house on solid bricks [24], then evidence can be compared to an anchor that solidifies the belief. Compared with positions teachers agree to, belief argumentations, such as providing reasons or evidence, seem to be more meaningful [25].

Another characteristic of belief systems, known as ‘clustering’, also relates to the subtle relationship between beliefs and practice. This refers to the way beliefs are held, rather than the content of beliefs. According to Green, belief systems consisted of relatively isolated small clusters, which can prevent the confrontation of certain beliefs and allow for seemingly conflicting beliefs to coexist [22]. These ‘conflicting’ beliefs are not contradictory to those holding those beliefs [4]. Different clusters may relate closely to different contexts [26,27]. A teacher may believe one thing or a group of things in one situation, and the opposite in another, demonstrating the contextualization of beliefs [13]. These apparent conflicting beliefs provide an important entrance into a deeper understanding of belief systems and a breakthrough for explaining the apparent inconsistency between teachers’ professed beliefs and their actual practices.

**Figure 1:**
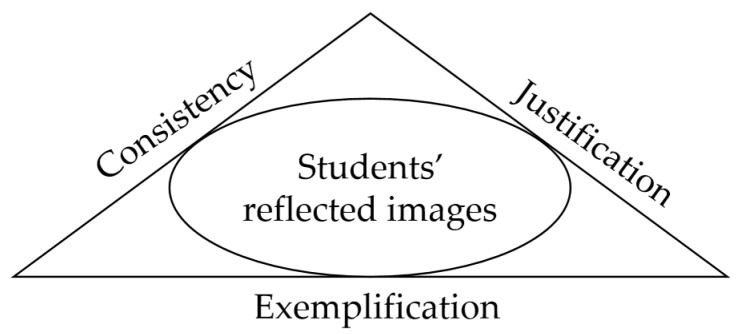
Students’ reflected images about mathematics as a discipline.

Although the quasi-logical structure, evidence-based nature, and clustering are overall characteristics of a belief system, the extent to which a belief relates to other beliefs, is grounded in evidence, and coheres with others varies. A reflective beliefs model was proposed to capture these differences, consisting of three dimensions: justification, exemplification, and consistency, shown in Figure 1 [28,29]. This model can be used to access and assess how beliefs are held. The concept of justification reflects the extent to which a belief can be justified—whether and to what extent a belief exists for a reason. In mathematics, the evidence supporting beliefs often takes the form of examples. For instance, examples of Shannon and Hamming’s work can be used to support the belief that research consists of ‘investigations and further development’ [28]. Here, knowledge about Shannon and Hamming’s work serves as evidence to support the belief, with examples playing a role in exemplification. The concept of exemplification is used to describe the ability to provide examples, or evidence in general, for their beliefs. The concept of consistency is used to describe and evaluate the degree of consistency between related beliefs or views.

Jankvist’s reflective belief model closely aligns with Green’s belief system, emphasizing how beliefs are held rather than their contents. Since this model focuses on the attributes of beliefs, and teachers’ beliefs share many similarities with students’ beliefs, it can offer insights into how teachers’ beliefs are held, even though the model is based on students’ data. However, there may still be differences between how beliefs about mathematics are held and how beliefs about mathematics teaching are held. Thus, the reflected belief model serves as a foundation for analyzing how teaching beliefs are held, with additional characteristics specific to beliefs about mathematics teaching being identified during data analysis.

## 3. Context and Methodology

This study is based on a one-year online in-service teacher education program in mainland China for junior middle school teachers focusing on the history and pedagogy of mathematics (HPM). The research team organized the program to develop teachers’ beliefs about mathematics and mathematics teaching. Teacher education programs are ideal for longitudinal studies of how teachers’ beliefs and practices develop over time [6]. In-service teachers were used because they had more plentiful teaching beliefs about mathematics after immersion in classroom teaching than pre-service teachers. Seventy-six in-service junior middle school teachers from 15 provinces of mainland China volunteered to participate in the program. Most of them came from economically developed and relatively developed areas of China. Although the version of the textbook used varies from province to province, the mathematics curriculums are all based on the national mathematics curriculum standards.

This program following a procedure for developing IHT (integrating the history of mathematics into teaching) cases, which included selecting a topic, preparing, designing, discussing, implementing, evaluating, analyzing, and writing [30]. Nine topics were selected, such as multiplication of negative numbers, the sum of angles in a triangle, representing numbers with symbols, isosceles triangles, and functions. All of these topics were part of the core curriculum in junior middle school. The history of these topics were plentiful; for example, there were many ways to explain why the multiplication of negative numbers is positive and to prove that the sum of angles in a triangle is 180°. Historically, there have been various definitions of functions and different approaches to representing numbers with symbols. Teachers studies the history of these mathematical concepts, along with teaching designs inspired by some expert mathematics teachers. They also had the opportunity to observe the students’ responses to the implemented teaching designs. Some teachers then used the history provided to design their own teaching. Through discussions with other teachers and expert teachers, teachers revised their original teaching design several times and finally implemented it in the classroom. The researchers helped the teacher who implemented the lesson to receive feedback from students through questionnaires. The teacher who implemented the lesson shared the classroom video and the students’ feedback to all the teachers.

The development of belief systems involved subtle relationships among various beliefs, so the case study method was chosen to trace the complicated related relationships. Multiple-case studies were usually regarded as more convincing than single-case studies. The multiple cases were used to confirm or extend the theoretical discovery, instead of inferring the general characteristics. The selection of multiple cases should obey replication logic, instead of sampling logic [31]. This is an exploratory study and does not require a high degree of certainty, so according to Yin, 2 or 3 cases are enough [31]. Three case teachers Haley, Lily, and Sven (pseudonyms) were selected. Their background information was shown in Table 1. These three case teachers stood out from the other teachers for the following reasons. On the one hand, they actively participated in all of the program activities, which made it more likely for their teaching beliefs to change. On the other hand, they showed obvious development in the way teaching beliefs were held. Along the program, reflection tasks about the teaching of specific topics were assigned. Seven teachers were picked, as they shared more information about beliefs of mathematics teaching. Follow-up interviews were conducted with these teachers, and three of them were finally chosen as cases, as they showed interesting ideas about the way their teaching beliefs were held. It is obvious that the selection of cases was not random, but information oriented [32]. The information-oriented cases were usually atypical or extreme, as they activated more basic mechanisms in the study context, here being the subtle change in belief system. The atypical case selection means that the generalization is limited; however, the force of example for cannot be denied [32]. In this study, the three case teachers could not represent the other teachers, but they provided in-depth insight into the different ways teaching beliefs were hold.

To address the research questions, various methods of data collection were used, including Likert and open questionnaires on teaching belief (pre- and post-test), reflection tasks about the teaching of each topic, summative reflection and follow-up interviews, etc. Most Likert questions were from existing studies [26,33,34], aiming to roughly gauge teachers’ positions on remembering and applying conclusions, understanding knowledge, and encouraging student exploration. The reflection tasks on each topic were general, such as “how do you usually teach this topic before the program? please talk about the change of your belief of the teaching of this topic in terms of teaching goal, teaching focus etc.”. These tasks aimed to elicit teachers’ beliefs related to specific topics; meanwhile, they were useful to know teachers’ teaching practices. The open-ended questionnaire aimed to elicit teachers’ beliefs of general mathematics teaching, which can form a triangulation of data together with reflection tasks on specific topics. Some questions were direct to their positions on the concept that the new curriculum advocated, such as ”Do you think your students should understand the context in which knowledge is produced? What do you think about cultivating your students’ positive mathematical emotion? What do you think about students’ independent exploration in mathematics teaching?” Some questions were more open and were from existing studies [3,35,36], such as “What’s the goal of your mathematics teaching? What’s the relative importance of various goals? What do you think is the role of teachers in mathematics teaching? What are the three most important characteristic of good mathematics teaching?” Targeted questions and open-ended questions can also form a triangulation of data. As this study aimed to explore changes in teaching beliefs, for each question in the post-test, teachers were asked to reflect their beliefs both before and after the program. The belief expressed in the pre-test and the reflection on the belief used to be held in the post-test formed a data triangle again. Also, as this study focused on the way beliefs were held; instead of just the content of beliefs, teachers were asked to illustrate the reason for their position on each question. In the summative reflection, teachers were asked to reflect on the influence of the program on their belief of mathematics teaching, which can elicit the most important change in teaching beliefs. The text could limit the in-depth information about the cases; therefore, some changes in beliefs showed in the questionnaires and reflection tasks were picked out to formulate questions of follow-up interviews. The case teachers were asked to explain more about the change in teaching beliefs, especially the details of the way teaching beliefs were held. Examples of interview questions were shown in Table 2. Two professors were invited to examine the questions in open questionnaire and interview outline and make suggestions for revision. Four semi-structured interviews were conducted for each case teacher throughout the program, and each interview lasted about 40 min. Using multiple methods of data collection allowed for the acquisition of rich information necessary for the research and enabled methodological triangulation. The triangulation can serve both as a method in itself (multiple lenses) and as a way to validate conclusions (multiple lines of evidence) [37].

How teachers held these beliefs before and after the program was coded deductively according to the dimensions of justification, exemplification, and consistency, as shown in Table 3. If certain characteristics of the way teaching beliefs were held could not be coded within these three categories, new categories were added inductively. Both top-down and bottom-up coding logic were employed during data analysis [38]. Two researchers, including the first author, independently coded the data from one case and reached a 96% agreement. The discrepancy codes were resolved through discussion.

## 4. Results

To address the research questions, we conducted an analysis of the evolution of belief systems for each case. We first provided more background information and then proceeded with a comparative analysis of these belief systems—both prior to and following the implementation of the teacher education program.

### 4.1. The Case of Haley

Haley dedicated a decade of her career to teaching at a rural high school before transitioning to a well-regarded secondary school. Prior to participating in the program, her understanding of the history of mathematics was confined to the content of two related lectures. During the program, Haley read 91% of the historical materials, participated in 83% of the online discussions, and submitted all the reflection tasks. She implemented the lesson “irrational numbers” and “the sum of the angles of a triangle”.

#### 4.1.1. Haley’s Beliefs and Practices before the Program

In the Likert questionnaire, Haley agreed with the statement, ‘Teachers should help students to like and cherish mathematics’. Improving students’ interest in mathematics was also one of Haley’s reasons for joining the program. However, Haley did not include fostering positive affect as a teaching goal in the open questionnaire. It appears she believed affective goals should be considered, but were of low importance to her. After participating in the program, Haley confirmed that she had not previously considered how to cultivate students’ interest in teaching practice.

Similarly, Haley initially agreed that self-exploration is important and marked ‘agree’ to the statement that teachers should encourage students to think about and discuss challenging problems. But after the program, Haley reflected that she had not understood why exploration was important before the program.

Before participating in the program, Haley had not paid much attention to exploration or the interest of her students. She held these beliefs because they aligned with the ‘common view’ of the educational community, not because she had reflected deeply about them. She did not consider their importance or how to implement them in her teaching. There was no justification provided, let alone exemplification. As a result, although Haley agreed that teaching should encourage exploration and cultivate students’ interest, she did not implement these ideas in her teaching practice. As Haley stated, she focused all her energy on teaching basic knowledge, skills, and exercises.

#### 4.1.2. Haley’s Changing Beliefs about Exploration

After the program, Haley believed that it was very important for teachers to give students plenty of time to explore problems by themselves. She emphasized the key role of appropriate problems or tasks for students’ exploration and believed that the nature of these tasks determined how much students could learn from them. More specifically, Haley believed tasks with various solutions were suitable for students to explore. For instance, she guided students to interpret why the product of two negative numbers is positive and why the sum of the angles in a triangle is 180° in different ways. The change in belief was related to the program. During the program, Haley learned different ways to explain the rule that the product of two negative numbers is positive and to prove the theorem that the sum of the angles in a triangle is 180° in history, which was the knowledge base that led students to inquire. More directly, she learned from other teachers that problem or task designs with various solutions can be used to encourage students to explore multiple problem-solving methods on their own.

These new beliefs are related to the execution of students’ exploration, not the importance or necessity of it. In other words, these beliefs are not reasons for holding the belief that students should have the opportunity to explore, but they support the latter belief. The new category ‘practicality’ was used to describe the extent of some beliefs about implementation.

**Figure 2:**
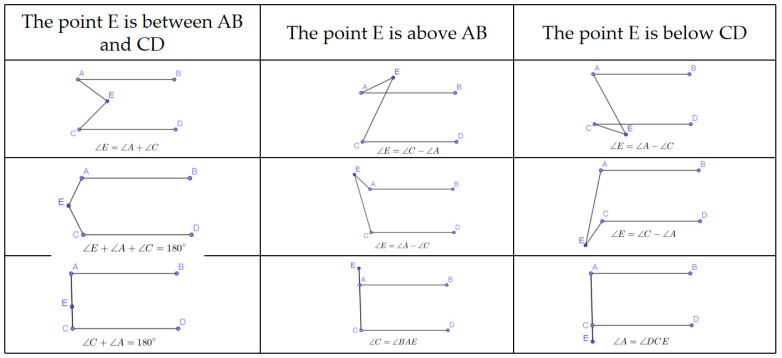
Different relationships among ∠E and ∠A and ∠C.

Haley justified the value of students’ exploration from different perspectives: it can stimulate students’ interest and improve students’ problem-solving abilities. She exemplified this with her teaching experience. In teaching the properties of parallel lines, Haley used a common exercise, as shown in the first figure in Figure 2, involving two parallel sticks and a rubber band.

AB and CD are two parallel sticks, and AEC is a rubber band with two ends fixed at two points, A and C. What’s the relationship between ∠E and ∠A, ∠C?

This simple problem could be solved in a few minutes, but Haley dedicated two classes to it, encouraging students to consider the situations if the rubber band was not dragged in the pattern shown in the first figure. In each situation, students were encouraged to find the relationship between ∠E, ∠A, and ∠C, and provide a proof. Haley adapted this routine question to make it open-ended and suitable for students to explore, which was a response to her belief that tasks with various solutions were suitable for students to explore. Eventually, the students discovered eight other different situations beyond the first one, as shown in Figure 2. The students actively engaged in and enjoyed the inquiry process, continuing to discuss relevant issues even after class. This experience helped Haley in recognizing students’ potential and the importance of divergent exploration in stimulating students’ motivation to learn.

Furthermore, Haley noted that her students performed well on the chapter test about parallel lines, which she attributed to their in-depth exploration of this problem. During this process, students studied various relationships between points and lines, so they could identify basic structures within complex graphs and solve related problems. While, good exam performance was not explicitly used to justify encouraging exploration, it remained a hidden but important reason, as strong exam results have always been a significant teaching goal for Haley.

#### 4.1.3. Haley’s Changing Beliefs about the Interests of Students

In addition to her evolving beliefs about exploration, Haley reported that her beliefs about improving students’ interest in mathematics changed the most during the program. She justified this belief by observing that students learned more actively when they were interested. More importantly, this belief was supported with practical examples as evidence.

As mentioned in the previous example about exploring the relationship between angles, Haley found that students will actively engage in problems that interest them, even outside of class time. More broadly, Haley shared her students’ enthusiasm for completing their homework:

*I have this experience because of my students. I did not leave much homework for them, but my students like to learn mathematics, and they bought books to do by themselves… The students also tell their parents that they learn many things but do not feel too tired. I think he will learn because he is interested in learning, so you don’t force him to learn.*

Similar to her justification for why exploration is important, another key reason for Haley to attach importance to students’ interest was the positive effect on learning outcomes. Even though Haley did not directly link the importance of interest with good exam performance, she implicitly expressed such a relationship.

Haley found that her students’ interest in learning heightened during the second half year of the program, and students’ average scores were ten points higher than those of the other class in the final exam. Therefore, Haley began associating students’ interest positively with good performance—a very important goal for her. Haley argued that too much compulsory exercise can lead to ‘half the effort’, while interest can promote ‘twice the effort’. In Haley’s belief system, exploration, interest, active engagement, problem solving, and good performance began to support and inform each other.

### 4.2. The Case of Lily

Currently, Lily teaches at a school that ranks top among the schools in the district. Prior to participating in this program, she was engaged in a research project focused on mathematics teaching. During her postgraduate studies, she was briefly acquainted with the history of mathematics. During the program, Lily read 96% of the historical materials, participated in 91% of the online discussions, and submitted 91% of the reflection tasks. She implemented the lesson “function”.

#### 4.2.1. Lily’s Beliefs and Practices before the Program

Before participating in the program, Lily believed that teachers should encourage students to learn through self-exploration and allocated more than half of the classroom time to exploration. She justified her approach by stating that exploration serves as means to cultivate students’ thinking, a goal she has consistently emphasized as important in teaching mathematics. She also argued that self-exploration enhances students’ understanding of the subject matter knowledge. Although Lily provided reasons from these two perspectives, her argument was theory-based and she did not offer any concrete examples to support her arguments.

There are some inconsistency beliefs in Lily’s belief system. While Lily maintained that exploration is a method to cultivate students’ thinking, she used to say that she did not know how to train thinking process, which suggests that Lily may not have given much consideration to the practical application of self-exploration. The practicality of her belief, therefore, seems weak. In the interview, Lily confirmed this assumption: she used to emphasize encouraging exploration simply to allow for more students’ discussion. Moreover, Lily expressed inconsistent beliefs regarding the relationship between exploration and exercise, as well as their impact on performance:

*I used to feel that there was a conflict. The class was so short, if you let the students do activities, then there would be less time left for exercise. As a result, the teaching task could not be thorough, and the students may perform poorly in the exam.*

**Figure 3:**
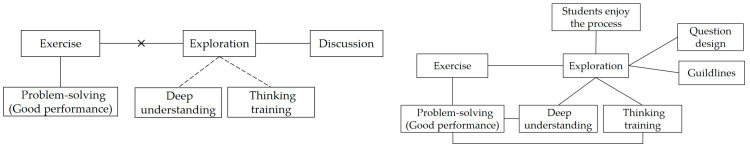
Lily’s metaphoric belief system before (**left**) and after (**right**) participating in the program.

Influenced by the former Soviet Union’ educator Kailov’s five-step teaching method, which include organizing teaching, reviewing the learned lesson, explaining the new lesson, consolidating the new lesson, and assigning homework, teaching in mainland China typically follows a structured flow, where examples and exercises are integral elements of every class [39,40,41]. Lily believed that teaching is incomplete without sufficient examples and exercises. In her view, regular exercise is crucial, as it prepares students for good performance, enabling them to solve problems in exams. As such, she did not encourage exploration activities that could consume a significant portion of class time in her teaching practice. For Lily, exercise and good performance are more important than exploration and its possible outcome, thinking training. Lily’s belief system relating to students’ exploration is described in the left figure of Figure 3. The connections between beliefs are represented by lines. Specifically, connections without supporting examples are indicated by dotted lines, while negative connections are denoted by an “×”.

There is a potential way to reconcile these inconsistent beliefs. Lily regarded thinking training as an important objective of learning mathematics in general. However, in the context of specific classes, she believed that if students could apply the acquired knowledge to complete exercises, her goal of teaching was achieved. In this way, the concept of “exploration–thinking training” and “exercise–good performance” can co-exist within two distinct clusters, which might be referred to as the “holistic and concrete” or “ideal and reality”.

#### 4.2.2. Lily’s Changing Beliefs about Exploration

Initially, Lily held a contradictory belief system: she valued exploration as a means of training students’ thinking; however, she was also concerned about classroom time, balancing exercises, and achieving high exam scores. This created a conflict between her ideal teaching methods and the reality of classroom constraints. Following the program, there was a substantial shift in Lily’s beliefs regarding exploration and practice in the classroom. Lily held a more consistent belief about encouraging students’ exploration. The contradiction between students’ exploration and exercise had weakened. One of the key reasons was that Lily no longer considered extensive practice as an essential part of the classroom.

Lily believed that if students could understand deeply, even without much exercise, the teaching task could also be considered completed. This belief was supported by the ‘authority’ of an expert teacher she admired, who once said during a discussion on function design: “Just make the students realize the function is the relation between two variables, the rest can be dealt later”. This comment eased her anxiety: initially, she wanted her students to experience the function as a specific relationship between two variables and demonstrated with many examples and exercises within 45 min. Acceptance of less practice freed up more time for student exploration. Due to the expert teachers’ teaching practices and discussion, she embraced a flexible classroom instead of a fixed routine.

Moreover, the positive effects of exploration also encouraged Lily to consistently promote this approach. She began to realize that deep understanding and flexible thinking, achieved through exploration, could be more beneficial than extensive practice for problem solving. Problem solving skills closely related to good exam performance, aligning with her goal of practice.

*I think my beliefs may have changed, you do not have to practice a lot, you explore deeply, (in the process) you think more and establish the knowledge network. After understanding the essence of one thing, one can infer other things of the similar kind by analogy, and solve some variant problems.*

*In fact, thinking training in each class is not in conflict with achieving teaching goals, and completing teaching tasks (problem solving). You train student’s thinking in the class, his mind will be open, and he can think more. Their mathematics ability will be stronger naturally.*

Lily often referenced “good teaching” practices observed in the program to illustrate her beliefs, such as a lesson on “the sum of the interior angles of a triangle”. In this lesson, the teacher encouraged students to prove that the sum of a triangle’s interior angles is 180° using various methods and the students do construct different auxiliary line to prove the theorem. Lily believed that such exploration facilitated the resolution of similar problems as students had in-depth thinking about the relationship between line segments and angles. Moreover, exploring different methods promoted flexibility in students’ thinking, a critical aspect of problem solving. So, expert teachers’ research lessons in the program, including the lesson design and the students’ performance, are the reason for Lily’s changing beliefs, and are used as examples by Lily to support her changing beliefs. The influence of expert teachers in the teacher education program significantly contributed to this shift. Their insights and practices offered her a fresh perspective on mathematics teaching and the fluidity of classroom routines.

Another reason Lily offered for advocating extended exploration time was that she believed students enjoy the exploration, which can be exemplified by her teaching experience. One of the important activities of the research lesson “function” was asking students themselves to conclude the commonalities of different relationships between two variables. Lily delighted in observing her students’ active engagement during exploration. Her students’ feedback, such as exclamations like “Really? Is the class over?”, reflected their enjoyment in the exploration too, which is an outcome Lily highly valued. This demonstrated the positive impact of exploration activities. Lily recognized the value in the enjoyment students derived from exploration, which amplified her appreciation for its implementation.

Before participating in the teacher education program, she primarily associated exploration with discussion. Post-program, she understood that exploration activities could be more varied, encompassing specific questions, focusing on key knowledge points, provision of guidelines, and a blend of problem-solving and exercise. Consequently, she perceived an enhancement in the practicality of her beliefs.

Figure 3 visualizes the evolution of Lily’s teaching beliefs regarding exploration before and after her involvement in the program. The practicality of belief became stronger, as Lily did not just equal the exploration as free discussion, but realized the importance of question or task design and the guidelines in effective exploration. Lily can justify the reason for exploration from more perspectives and provide examples. She also reconciled the conflict between practice and inquiry. Lily’s experience highlights the significance of continuous professional development and reflective practices in the field of education.

### 4.3. The Case of Sven

Sven holds a leadership role within his teaching group at his current school, which ranks in the upper-middle tier. His teaching career spans experience in both town schools and top-ranked institutions. Sven is deeply engaged in pedagogical research, having published papers on teaching methodologies and participated in several projects prior to his involvement in this teacher education program. His enthusiasm for the history of mathematics is notable, as reflected in his voluntary reading of books related to the subject. During the program, Sven read all the historical materials, participated in all the online discussions, and submitted all the reflection tasks. He implemented the lesson “mathematical axiomatization—isosceles triangle”, “use letters to represent numbers” and “the multiplication of rational numbers”.

#### 4.3.1. Sven’s Beliefs and Practices before the Program

Sven strongly believed in the necessity of contextualizing knowledge—explaining how the knowledge originated and developed over time. He identified this as a key motivation for his participation in the teacher education program and as an attribute of effective teaching. Sven substantiated these beliefs with his observations of students’ keen interest in the historical progression and development of mathematical topics:

*When I tried to do this (telling the context of the knowledge), they (students) always listened with full attention. Sometimes when I talk about some topic, they always ask me, ‘Sir, can you help us with the history of this topic?’*

**Figure 4:**
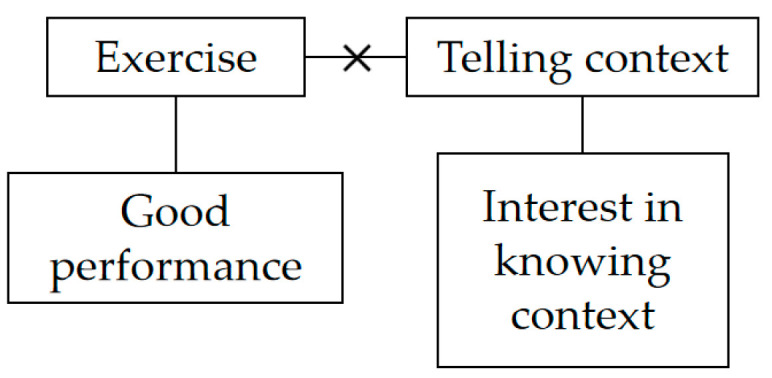
Sven’s metaphoric belief system before participating in the program.

However, Sven also held conflicting beliefs, as shown in Figure 4. He expressed concern that devoting excessive time to contextualizing knowledge could encroach upon the time allocated for practice, potentially influencing students’ performance. This belief stemmed from his firm conviction that consistent practice is crucial for achieving good exam performance, a goal he regarded as important. Due to these conflicting beliefs, Sven did not consistently incorporate the contextualization of knowledge into his teaching practice.

In addition to contextualization, Sven acknowledged the importance of student exploration. He posited that student-led exploration should supersede teacher-guided instruction:

*Teachers should design and organize the whole process of teaching, point and guide students’ thinking obstacles, and time spend on this should not exceed 30%. Students are the main part of learning, so we should leave enough time for students to explore, practice, question and solve problems.*

Despite this belief, Sven also expressed reservations about student exploration, highlighting the difficulties and challenges in organizing such activities:

*This is also a dilemma. If your design is not very appropriate, you may finally spend time to explore but get nothing… Organizing exploration activities is very demanding. Teachers should be able to teach everything to the students appropriately, with the most classical language, at the most appropriate time, to guide them and not tell him.*

Finally, Sven agreed that teachers should help students develop a passion for mathematics, but he did not prioritize this goal. He admitted:

*I did not pay much attention to this matter (students’ interest) before. I did not think much about the necessity and did not know how to fulfill it.*

Prior to participating in the program, Sven held several beliefs about teaching, such as the importance of contextualizing knowledge, promoting student exploration, and fostering a love for mathematics. However, these beliefs were not consistently implemented due to a lack of reflection, specifically, conflicting beliefs, poor justification and exemplification, and low practicality.

#### 4.3.2. Sven’s Changing Beliefs about Context

Sven consistently believed in the importance of explaining the context of mathematical knowledge. The program assisted in resolving the perceived conflict between providing context and striving for high scores, with “interest” playing a significant role:

*I used to worry that if I provide [the context], it would affect practice time, and by extension, the score. But now I don’t believe it would. On the contrary, they are positively correlated… I don’t mind reducing a few minutes of practice time. I might even devote some practice time to sharing historical context. I think that once interest is aroused, the subsequent teaching and learning process will become smoother.*

Before participating in the program, Sven justified and exemplified the need for context because students showed interest in it. His students displayed an interest in learning the history of mathematics. However, this was largely confined to a broad overview, inclusive of anecdotes and stories, which did not directly enhance exam performance. After attending the program, Sven maintained that providing context could stimulate students’ interest in learning mathematics. He backed this belief with examples: after he introduced and analyzed Proposition 5 in Euclid’s Elements (the proof for ‘in isosceles triangles, the angles at the base are equal to one another’), students expressed their admiration for mathematics. One of his students wrote, “It’s like the clouds parting to reveal the sun; I suddenly feel enlightened and appreciate the charm and value of geometry”. Certain students proactively compared and analyzed proofs in textbooks and Euclid’s Elements, thereby illustrating the axiomatic ideas of mathematics. One student’s paper on the topic was even published in a journal. This was not an isolated incident. Motivated by the history of mathematics, many students sought relevant materials for further study after school, with several publishing papers on topics such as ‘Analysing the Method to Calculate the Height of an Object That Is Inaccessible at the Bottom in Ancient China’.

Sven found that interest in learning mathematics positively impacted scores. He observed that students who engaged in micro-studies experienced an increase in their scores. One student noted in a mathematics essay: ‘Now that I like math, can improving math scores be that difficult?’ Therefore, Sven’s belief in explaining context gained significance because it correlated positively with the crucial teaching objective of achieving high scores, mediated by students’ interest. His belief in fostering students’ interest also grew in importance. Sven added students’ interest to the list of most important objectives, alongside basic knowledge and skills.

In addition to enhancing students’ interest in learning mathematics, Sven also proposed that explaining the context of mathematics could promote a deeper understanding of the subject and improve problem-solving abilities. He substantiated this claim with examples from his own teaching experiences. For instance, after introducing and analyzing Proposition 5 from Euclid’s Elements, Sven’s students developed a profound appreciation for the essence of mathematical axiomatisation. They noted that ‘basic facts are the foundation for developing theorems and the bedrock of geometric constructions’, and posed thought-provoking questions such as ‘Are basic facts immutable? What role does axiomatization play?’.

**Figure 5:**
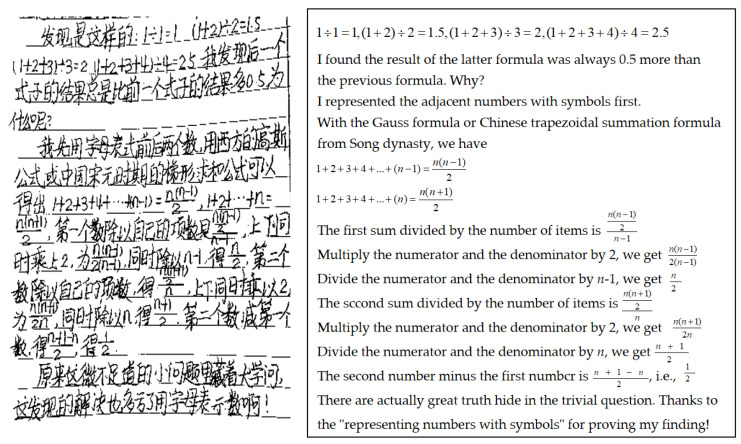
Proving mathematical law with symbols in Sven’s student’s work.

In another instance, while teaching the concept of ‘representing numbers with symbols’ was a research topic in the program, Sven guided students to appreciate the significance of this practice by contrasting ancient and modern mathematical approaches. Over time, one student proved a rule he had identified within numeric symbols in a math writing exercise (as shown in Figure 5.), thereby underscoring the value of representing numbers with symbols. This specific example illustrates how insights derived from the context of knowledge can facilitate problem solving. In general, during an interview, Sven justified the importance of providing context to learning:

*If we can present students with the context of knowledge, they will be able to comprehend it from a higher perspective. This level of understanding cannot be achieved by merely executing exercises mechanically, regardless of quantity. The context of knowledge will subtly influence students’ viewpoints, thought processes, and problem-analysis strategies in the future.*

**Figure 6:**
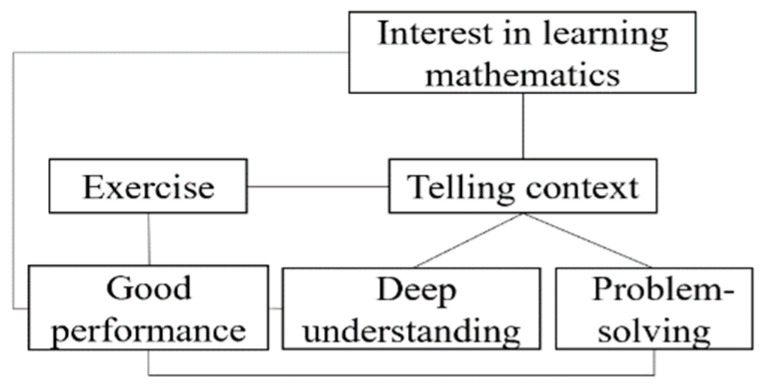
Sven’s metaphoric belief system after participating in the program.

Deep understanding and problem-solving abilities are crucial to improving students’ exam scores. Therefore, Sven’s belief in the importance of providing context evolved to positively correlate with his core belief in striving for high scores. Despite the potential for contextual teaching to reduce practice time and possibly impact scores negatively, Sven believed that providing context of mathematical knowledge could also improve scores from different perspectives. Figure 6 visually illustrates the change in Sven’s teaching beliefs about providing context after his participation in the program.

#### 4.3.3. Sven’s Changing Beliefs about Exploration

Sven’s belief in encouraging students’ independent exploration also intensified. He suggested that student-led inquiry could yield ideas and methods deserving further investigation, thereby contributing to a generative curriculum.

*Students’ independent inquiry can offer a wealth of research material for our classroom, potentially revealing valuable ideas and methods.*

Sven provided examples where, upon being given the freedom to explore, students uncovered effective methods, such as in teaching ‘the sum of the interior angles of a triangle’. While learning this theorem, students explored why the sum equals 180 degrees, and discovered numerous effective methods.

This approach implicitly recognizes students’ methods or ideas as valuable content, encouraging Sven to provide opportunities for students to generate and articulate their thoughts. Sven believed that students’ ideas might mirror those of past mathematicians, a notion referred to as historical parallelism.

*After the program, I believe we must pay attention to every student’s thinking, and not readily dismiss seemingly complex thoughts and methods. These approaches might mirror methods that humanity has utilized throughout the history of understanding mathematical problems.*

The value in students’ ideas lies in their potential to mirror those once proposed by esteemed mathematicians. This belief subtly reinforces the encouragement of student exploration. Within a belief system, a specific belief may be consciously supported by certain arguments, but it can also be subtly or unconsciously fortified by other underlying beliefs. The term ‘connection’ is an inductive category, reflecting the extent to which a belief is supported by other beliefs that may not have been explicitly reported as reasons by the belief holder.

## 5. Discussion

There was an apparent inconsistency between the case teacher’s beliefs and practices before the program. It was explained that some professed beliefs are more-so a manifestation of a verbal commitment to abstract ideas about teaching [2]. This study provided an in-depth portrait of how beliefs as a verbal commitment were held by individuals and further examined how this kind of belief can be held in different ways.

**Figure 7:**
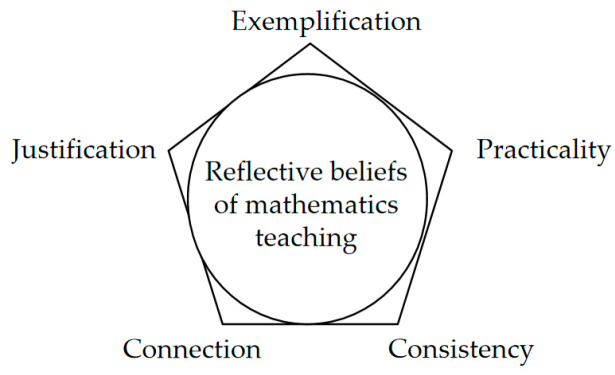
A reflected beliefs model of mathematics teaching.

Based on the analysis of how beliefs about mathematics teaching were held before and after the program, a reflective beliefs model of mathematics teaching is proposed (see Figure 7). This model allows for a more nuanced analysis of the way teaching beliefs are held. It encompasses five dimensions: exemplification, justification, connection, practicality, and consistency. Exemplification involves teachers illustrating their beliefs with specific examples. Justification involves demonstrating the rationality of their beliefs. Connection involves associating one belief with others positively, but not as reasons. Consistency involves holding a certain belief consistently in various contexts. Practicality involves having propositions on how to implement a belief in teaching practice. These five aspects can be regarded as the definition and explanation of reflective mathematics teaching beliefs.

Exemplification, justification, and consistency are already present in Jankvist’s model [28]. However, one key difference is that examples for beliefs about mathematics often relate to knowledge (such as changes in the definition of a function). In contrast, examples for beliefs about mathematics teaching often relate to teachers’ teaching experiences, primarily student feedback, including learning outcomes and processes. For instance, Haley exemplified the belief that interest drives independent learning, with her students doing more homework voluntarily. Sven exemplified the belief that context prompts deep understanding, with his students gaining deep insights into axiomatization due to proposition 5 in Euclid’s Elements. The change in Lily’s belief showed that other teachers’ teaching practice and their students’ feedback can also become the material for teachers’ reflection, and then affect the way beliefs were held. It should be noted that the other teacher here is a teacher Lily knows well and admires. The general condition that one’s experiences influence others’ belief needs further investigation.

Teachers’ reform-oriented beliefs, such as explaining the context of knowledge and encouraging inquiry activities, were inconsistent, which is mainly because such activities were believed to be conflicted with exercises. Teachers themselves did not feel as if they struggled, as they had their own way to reconcile them. First, the conflicting beliefs differed in psychological strength. Sufficient exercises were conducive to problem solving and good academic performance, which is a more important implicit teaching goal. Second, they may exist in different cluster called “holistic and concrete” or “ideal and reality”. Teacher education programs that aim to challenge teachers’ beliefs need to help teachers to reconcile these beliefs.

Connection is a new dimension in the model that reveals the extent to which other beliefs subtly support a belief, not in a direct reason–conclusion manner. The quasi-logical structure between beliefs has been referred to by Green [22]. Some beliefs act as reasons to support a certain belief, characterized as justification in the reflective belief model. Some beliefs connect positively with a certain belief, supporting the reason for that belief. In this case, if you ask, “why do you believe this”, they do not emerge. For instance, Sven argued that exploration should be encouraged because valuable ideas can be generated. He never used the reason “students’ ideas should be appreciated as those may be the ideas of mathematicians”. However, the belief that “students’ ideas are worth being valued” does support the belief “exploration should be encouraged”. Another example is a teacher who supported using technology in teaching, and one of the reasons was that it was helpful to achieve better understanding. The belief “mathematics as a discipline is rather ‘experimental’ in nature” also supports this belief, although for not as a direct reason [42]. If there is a longer chain of syllogisms, a belief can relate to more beliefs into a larger system, and others are interrelated into smaller systems [43]. The more a belief is connected to other beliefs in a vertical structure way, the more central a belief may be, as they have roots deep in the belief system.

Practicality is another new dimension, indicating if and to what extent there are beliefs related to teaching practice, including the implementation, or manifestation of a belief in practice. Considering practicality is helpful to investigate the relationship between beliefs and practice. This aspect has been emphasized in research exploring beliefs that guide classroom practices or personal practical theories [44], responding to the association between beliefs and practical knowledge [45]. Before participating in the program, Haley and Lily had no views on stimulating interest or organizing exploration activity. They could abstract the characteristics of tasks suitable for exploration after the program. Reform-based teaching practice is an important driving force of belief change [46], so as the evolution of belief occurred, the practicality of beliefs was enhanced. High practicality of beliefs comes from reflecting on one’s own or others’ teaching practice related to that belief. Only if a belief is relevant for a range of behaviors can it be more central [43]. No one emphasizes a teaching belief he or she has no idea how to implement.

As we know, changes in teachers’ beliefs need a comparatively long period. Compared to the content of beliefs, the way beliefs were held can be changed in a relatively short period of time through reflection on new experience. In this study, teachers rarely rejected their original beliefs, they just reinforced their beliefs with examples, reasons, and implementation method, as well as reharmonized the contradictory relationship among beliefs. In fact, these changes in the way beliefs were held can also be termed as the change of “conception” or “perspective”. “Conception” was viewed as a more general mental structure than “beliefs” [2]. However, based on the adopted conceptual framework, mainly Green’s belief systems, the term “beliefs” was used in this study. In Rodríguez-Muñiz et al. [47], the interrelatedness of beliefs and conceptions were emphasized, instead of the distinctions.

This study found that the students’ positive feedback and the teaching practices that produced this feedback played a vitally important role in challenging the way beliefs were held. This finding reinforced the statement that noteworthy outcomes from professional experimentation have been recognized as integral to teacher development [48,49]. Students’ positive feedback evoked teachers’ positive emotions, like surprise, joy, and pride, which seems quite helpful to promote teachers’ belief change.

Although a variety of data sources were used to reveal teachers’ beliefs systems in this study, the data were mostly personal verbal reports and the beliefs were professed beliefs. Teachers’ teaching practice before the program was also based on teachers’ oral descriptions rather than classroom observations. Teaching practice was not made full use of in order to tap into teachers’ underlying beliefs, which is a limitation of this study. Based on the assumption that a teacher’s belief system is sensible system, exploring the relationship between belief and practice facilitates a deeper understanding of the nature of beliefs and how they are held [2,13].

## 6. Conclusions

This study addressed the apparent discrepancies between teachers’ beliefs and their teaching practices. Before the program, all three teachers held positive beliefs about providing context, encouraging exploration, and fostering interest, but rarely implemented them in teaching practice. These belief positions, often regarded as the ‘common view’ of mathematics educators, might have been expressed simply because they were expected. From the perspective of the way beliefs were held, teachers reflected little on these beliefs. It is almost impossible for a teacher to implement a belief that is not important and which faces important conflicting beliefs. We delved into the foundational belief structures of mathematics teachers and analyzed their evolution over a professional development program. The findings resulted in developing a unique model that encapsulates five dimensions of reflective mathematics teaching beliefs: exemplification, justification, connection, practicality, and consistency. It is hard to guarantee that a belief with high degree of these five dimensions must be implemented in practice, but it is more possible to be implemented. So, the reflected beliefs model of mathematics teaching is useful to examine the relationship between belief and practice, both in the direction of what kinds of beliefs are more likely to influence practice, and in the direction of how practice influences beliefs.

While it is derived from mathematics teachers’ cases, the reflective model proposed in this study is not confined to that subject. It could offer a more nuanced framework for assessing teachers’ beliefs and could guide teachers towards more directional reflection on their subject’s teaching beliefs. This broad applicability underscores the model’s potential to influence what teachers believe and how they believe it, addressing a significant challenge in teacher education [13]. The mechanisms underpinning how mathematics teachers hold their teaching beliefs were explored in this study; the difference in the way beliefs were held and the characteristic of the change in beliefs for different teacher groups can be explored in the finer-grained level in the future.

This study shines a light on the intricate structures of teachers’ teaching belief systems by proposing the reflected beliefs model of mathematics teaching. This study also offers a new perspective for understanding and improving teaching practices. Our findings underscore the importance of fostering reflective practices in teacher education programs to promote the alignment of teachers’ beliefs with their teaching practices. By understanding and applying this model, educators can integrate and embody their teaching beliefs more effectively, leading to more consistent and effective teaching practices. This, in turn, can result in better student learning outcomes and contribute to the overall improvement of education quality.

## Figures and Tables

**Table 1:** Case teachers’ background information.

	Gender	Teaching Experience (Years)	Teaching Grade	Education Experience
Sven	Male	19	9	Bachelor of mathematics
Haley	Female	15	7	Bachelor of mathematics
Lily	Female	6	8	Bachelor of education and Master of mathematics education

**Table 2:** Examples of interview questions.

Case	Text (Questionnaires and Reflection Tasks)	Interview Questions
Haley	The biggest change is that [my teaching] is less utilitarian.	Can you explain in detail of this change? How did the change happen?
Lily	Let the students really be the masters of their learning, and let students experience the process of concept formation more by themselves.	How did the change happen?
Sven	I didn’t pay much attention to students’ independent inquiry before.	Why didn’t you pay much attention to students’ independent inquiry before?

**Table 3:** Coding framework.

Category	Definition	Example
Justification	Justify some belief with reasons for the importance, necessity, etc.	We should attach importance to students’ independent inquiry, because independent inquiry can stimulate learning interest.
Exemplification	Support some belief with examples of knowledge, experiences, etc.	Independent inquiry can stimulate students’ learning interest. For example, once students explored (a certain issue) independently in class, they still enjoyed the exploration and continued to discuss even after class.
Consistency	Different beliefs are consistent and coherent, without contradiction and conflict.	Inquiry activities should be encouraged, but it conflicts with practice as it takes a long time. (Counterexample)

## Data Availability

The datasets used and/or analyzed during the current study are available from the corresponding author upon reasonable request.
